# The changing role of substances: trends, characteristics of individuals and prior healthcare utilization among individuals with accidental substance-related toxicity deaths in Ontario Canada

**DOI:** 10.1371/journal.pone.0324732

**Published:** 2025-05-23

**Authors:** Shaleesa Ledlie, Alice Holton, Pamela Leece, Bisola Hamzat, Joanna Yang, Gillian Kolla, Nikki Bozinoff, Rob Boyd, Mike Franklyn, Ashley Smoke, Paul Newcombe, Tara Gomes

**Affiliations:** 1 Li Ka Shing Knowledge Institute, St. Michael’s Hospital, Toronto, Ontario, Canada; 2 Leslie Dan Faculty of Pharmacy, University of Toronto, Toronto, Ontario, Canada; 3 ICES, Toronto, Ontario, Canada; 4 MAP Centre for Urban Health Solutions, St. Michael’s Hospital, Toronto, Ontario, Canada; 5 School of Pharmacy and Biomolecular Sciences, RCSI Ireland, Dublin 2, Ireland; 6 Public Health Ontario, Toronto, Ontario, Canada; 7 Dalla Lana School of Public Health, University of Toronto, Toronto, Ontario, Canada; 8 Department of Family and Community Medicine, University of Toronto, Toronto, Ontario, Canada; 9 Faculty of Medicine, Memorial University, St. John’s, Newfoundland and Labrador, Canada; 10 Campbell Family Mental Health Research Institute, Centre for Addiction and Mental Health, Toronto, Ontario, Canada; 11 Ottawa Inner City Health Inc, Ottawa, Ontario, Canada; 12 Northern Ontario School of Medicine, Sudbury, Ontario, Canada; 13 Ontario Drug Policy Research Network Lived Experience Advisory Group, Toronto, Ontario, Canada; 14 Institute for Health Policy, Management and Evaluation, University of Toronto, Toronto, Ontario, Canada; Xiamen University - Malaysia Campus: Xiamen University - Malaysia, MALAYSIA

## Abstract

**Objective:**

To investigate trends and the circumstances surrounding fatal substance-related toxicities directly attributed to alcohol, stimulants, benzodiazepines or opioids and combinations of substances in Ontario, Canada.

**Methods:**

We conducted a population-based cross-sectional study of all accidental substance-related toxicity deaths in Ontario, Canada from January 1, 2018 to June 30, 2022. We reported monthly rates of substance-related toxicity deaths and investigated the combination of substances most commonly involved in deaths. Demographic characteristics, location of incident, and prior healthcare encounters for non-fatal toxicities and substance use disorders were examined.

**Results:**

Overall, 10,022 accidental substance-related toxicity deaths occurred, with the annual number of deaths nearly doubling between the first and last 12 months of the study period (N = 1,570–2,702). Opioids were directly involved in the majority of deaths (84.1%; N = 8,431), followed by stimulants (60.9%; N = 6,108), alcohol (13.4%; N = 1,346) and benzodiazepines (7.8%; N = 782). In total, 56.9% (N = 5,698) of deaths involved combinations of substances. Approximately one-fifth of individuals were treated in a hospital setting for a substance-related toxicity in the past year, with the majority being opioid-related (17.4%; N = 1,748). Finally, 60.9% (N = 6,098) of people had a substance use disorder diagnosis at time of death.

**Conclusions:**

Our study shows not only the enormous loss of life from substance-related toxicities but also the growing importance of combinations of substances in these deaths. A large proportion of people had previously interacted within an hospital setting for prior substance-related toxicity events or related to a substance use disorder, representing important missed intervention points in providing appropriate care.

## Introduction

The rate of substance-related toxicity deaths continues to dramatically increase across Canada, with the number of opioid-related deaths attributable to fentanyl in combination with cocaine and methamphetamine increasing 39.2% (564–785) and 73.6% (519–901), respectively between 2018 and 2022 [1]. Further, over half of all deaths attributable to opioids over this period occurred among people aged 30–49 years [1]. Different factors have likely exacerbated the drug toxicity crisis over the past few years, including the increasing potency and volatility of the unregulated opioid drug supply (primarily composed of fentanyl and its analogues) and various societal changes which occurred during the COVID-19 pandemic reduced access to harm reduction programs, and increased anxiety and uncertainty resulting in changing patterns of substance use [2–5]. While opioids have dominated the discussion around substance-related harms across Canada in recent years, there is an increasing emphasis on the harm attributed to other substances such as benzodiazepines, stimulants, and alcohol, either alone or in combination with opioids. For example, during the COVID-19 pandemic, a rise in alcohol consumption levels was recorded [6], which coincided with a parallel increase in alcohol-related deaths resulting from both acute toxicities and longer term complications such as liver disease [7,8]. Further, although toxicity deaths directly attributed to benzodiazepines alone are less commonly reported, evidence of the introduction of these substances (such as etizolam) within the unregulated drug supply over the past five years has raised concerns given the established risks of combined use of opioids and benzodiazepines [9–11].

The rising prevalence of multiple substance use has been described as a ‘fourth wave’ in the overdose crisis [12], with data from Ontario showing that psychostimulants (such as cocaine and methamphetamine) contributed to 58.1% of opioid-related toxicity deaths in Ontario during the COVID-19 pandemic [13]. The unintentional use of multiple substances may also occur when a person consumes a substance that has been mixed or cut with additional substances (such as fentanyl mixed with benzodiazepines) [14]. This is especially concerning given that opioids combined with benzodiazepines or alcohol can an increase the risk of respiratory depression and sedation, while the use of opioids and stimulants in combination is postulated to increase risk of cardiovascular and cerebrovascular strain, both resulting in a higher risk of substance-related toxicity and mortality [15,16]. The use of multiple substances such as opioids, stimulants, benzodiazepines and alcohol can also complicate overdose response, particularly when multiple sedating substances are used together [17].

Despite this, research across Canada has largely focused on opioid-related toxicity deaths, with data still limited on deaths attributable to alcohol, benzodiazepines and stimulants. It is vital to understand the changing role of different substances in toxicity deaths and the circumstances around these deaths to help develop and inform community-based programs, access to treatment services and harm reduction interventions [18]. As a result, we aimed to investigate trends and circumstances surrounding fatal substance-related toxicities due to alcohol, stimulants, benzodiazepines or opioids in Ontario, Canada, including the changing role of multi-substance involvement, prior substance use disorder diagnoses, and previous hospital-treated non-fatal substance-related toxicities.

## Methods

### Study setting and design

We conducted a population-based repeated cross-sectional study of all accidental toxicity deaths where alcohol, stimulants, benzodiazepines or opioids directly contributed to death in Ontario, Canada from January 1^st^ 2018 to June 30^th^ 2022. We defined a substance-related death as an accidental acute toxicity death which resulted from the direct contribution of the consumed substance, regardless of how that substance was obtained (i.e., both pharmaceutical and non-pharmaceutical use). We excluded deaths that occurred among individuals without a valid Ontario health card for whom data linkage was not possible. The study is reported as per the Strengthening the Reporting of Observational Studies in Epidemiology (STROBE) guidelines [19] with a preliminary analysis of this data previously made available online [20,21].

### Data sources

We obtained all data for this analysis from ICES, formerly known as the Institute for Clinical Evaluative Science, an independent, non-profit research institute whose legal status under Ontario health information privacy law allows it to collect and analyse healthcare and demographic data without consent, for health system evaluation and improvement. We first accessed this data on July 13, 2023. Coroners in Ontario investigate all sudden and unexpected deaths. For this study, we used two datasets from the Office of the Chief Coroner of Ontario/Ontario Forensic Pathology Service. We used the Drug and Drug/Alcohol Related Death database to identify confirmed opioid toxicity deaths (with or without other substance involvement) and a newly derived database that includes data on alcohol, stimulant and benzodiazepine related-deaths where opioids did not directly contribute to death to support complete capture of all substance-related toxicity deaths in Ontario involving at least one of these four substances. Sociodemographic characteristics and population denominators were captured using the Registered Persons Database. We used the Canadian Institute for Health Information’s National Ambulatory Care Reporting System and Discharge Abstract Database, the Ontario Mental Health Reporting System and the Ontario Health Insurance Plan to identify emergency department (ED) visits, acute hospital admissions, mental health related hospital admissions and outpatient visits for substance use disorders. All the above datasets were linked using anonymized unique encoded identifiers and analysed at ICES. The use of the data in this study is authorized under Section 45 of Ontario’s Personal Health Information Protection Act (PHIPA) and does not require review by a Research Ethics Board.

### Trends and characteristics of individuals who died from a substance-related toxicity

Over the study period, we reported monthly population-adjusted death rates (per 100,000 population) directly attributable to each of alcohol, stimulants, benzodiazepines and opioids (these groups were not mutually exclusive as substance toxicity death may be attributed to more than one substance group). We stratified monthly rates of overall substance-related toxicity deaths by the number of substances directly contributing to death (1, 2, or 3+). We identified all combinations of substances directly contributing to death over the study period and determined the most common substance combinations.

We summarized demographic characteristics of all individuals who died of a substance-related toxicity over the study period, including age (grouped as: < 25, 25–44, 45–64 and ≥65 years old),[22] sex, neighborhood income quintile and location of residence (urban/rural and Northern/Southern Ontario). We also evaluated circumstances surrounding deaths, including the location of incident, and the type and number of substances directly contributing to death. Finally, we summarized prior healthcare encounters before death (S1 Table), including hospital encounters for non-fatal substance-related toxicities in the past year and substance use disorder diagnoses identified during hospitalizations (both any diagnosis type and most responsible diagnosis type) or outpatient physician visits (S2 Table).

### Statistical analyses

To examine the impact of the COVID-19 pandemic-related state of emergency on the trends of substance-related toxicity deaths over time, we used a combination of Joinpoint regression and ARIMA models [23]. Joinpoint regression models are recommended in situations where there is no pre-identified notion of where breakpoints in trend data may occur and can be used in the analysis of any outcome assessed over equally distributed time points [24]. Prior research has shown that the impact of the COVID-19 pandemic on rising rates of substance-related deaths began to plateau in late 2020 and early 2021 [25,26], however it was not possible for us to determine *a priori* the time at which this change in trend would have started. The Joinpoint software identifies points where a trend significantly changes, using the simplest model the data will allow [27]. We used the recommended Grid Search method [28] to select the best fitting Joinpoint model for our data. Using this model, we identified one monthly time-point (August 2020) which represented a significant breakpoint following the onset of the pandemic.

We considered this time-point as an intervention and conducted an ARIMA analysis to simultaneously model the onset of the COVID-19 pandemic-related state of emergency in March 2020 and this additional intervention point. We first plotted our monthly data to visually examine trends with our two intervention points to check for gradual or immediate changes. We determined that the onset of COVID-19 and the additional intervention point in September 2020 should both be fit using a ramp transfer function due to the gradual impact on death rates. As such, we did not consider pulse and step functions. Our ramp transfer functions began in March and September 2020, respectively and increased by a value of one each month until the end of the study period [29]. Our parameter estimates therefore represent the average change in the rate of substance-related toxicities each month. We determined that seasonal differencing was required to achieve stationarity, confirmed with the Dickey-Fuller test to ensure the unit root was removed from our time series. Although seasonal ARIMA (SARIMA) models have been shown to improve forecasting accuracy in the presence of strong seasonality [30–32], this was not the focus of our analysis. To minimize model complexity and reduce the risk of overfitting [33,34], we selected an ARIMA model with seasonal differencing. We used the residual ACF, partial autocorrelation function, and inverse autocorrelation function plots to select appropriate model parameters. Model diagnostics including Akaike’s Information Criteria (AIC), Bayesian Information Criteria (BIC) and the auto-correlation plots were used to select our final model parameters (p, q, d) (S3 Table). We used the Ljung-Box chi-square test for white noise to ensure that there was no significant autocorrelation in model residuals.

Finally, to test for changes in the prevalence of combinations of substances directly contributing to toxicity deaths between the first and last 12 months of the study period, we conducted chi-square tests using a type 1 error rate of 0.05 to define statistical significance. All analyses were conducted using SAS version 9.4 (SAS institute, Cary, North Carolina, USA) and Joinpoint Trend Analysis Software from the National Cancer Institute website (Version 4.8.0.1).

### Involvement of people with lived experience

The Ontario Drug Policy Research Network’s opioid-related research is informed by a Lived Experience Advisory Group, comprised of individuals with lived and living experience using opioids who were consulted before, during, and after the study design process. Subsequently, we identified several additional people with lived and living experience with a range of substances including opioids, stimulants, benzodiazepines, and alcohol. Throughout the conduct of this study, these individuals participated as study team members, providing feedback on the study scope, methodology, contextualization, and reporting of results. All people with lived and living experience were compensated for their time spent on this study and were offered co-authorship (or when preferred by the individual, acknowledgment) in this manuscript.

## Results

### Characteristics of individuals

Over the study period, we identified 10,785 substance toxicity deaths of which 10,022 (92.9%) met our inclusion criteria. Among these 10,022 substance-related toxicity deaths, opioids directly contributed to the majority of deaths (84.1%; N = 8,431), followed by stimulants (60.9%; N = 6,108), alcohol (13.4%; N = 1,346) and benzodiazepines (7.8%; N = 782; Table 1). Overall, 74.9% (N = 7,505) were among males, approximately half were aged 25–44 (51.9%; N = 5,197), and 37.6% were aged 45–64 (N = 3,768). The location of incident was recorded for 9,892 individuals, with the majority occurring at a private residence (72.8%; N = 7,298), followed by other indoor spaces [excluding hospitals] (8.2%; N = 820), outdoors (7.2%; N = 720), and rooming houses or collective dwellings (5.9%; N = 596). Overall, deaths were skewed towards neighbourhoods with the lowest income quintile (40.9%; N = 4,101). While the majority of deaths occurred in urban areas (89.3%) and Southern Ontario (86.9%), the rate of substance-toxicity deaths in Northern Ontario was almost three times higher compared to Southern Ontario (47.9 per 100,000 compared to 16.9 per 100,000, in 2021), and rates were similar between urban and rural areas (18.5 per 100,000 and 17.0 per 100,000, respectively).

**Table 1:** Descriptive characteristics, overall substance-related toxicity deaths.

	N (%) N = 10,022
**Substances contributing to death** ^*^	
Opioids	8,431 (84.1%)
Stimulant	6,108 (60.9%)
Alcohol	1,346 (13.4%)
Benzodiazepine	782 (7.8%)
**Age, median (IQR)**	41 (32-52)
<25 years	736 (7.3%)
25 to 44 years	5,197 (51.9%)
45 to 64 years	3,768 (37.6%)
≥65 years	321 (3.2%)
**Sex**	
Male	7,505 (74.9%)
Female	2,517 (25.1%)
**Income Quintile**	
Q1	4,101 (40.9%)
Q2	2,189 (21.8%)
Q3	1,539 (15.4%)
Q4	1,107 (11.0%)
Q5	930 (9.3%)
Missing	156 (1.6%)
**Location of residence**	
Urban	8,951 (89.3%)
Rural	916 (9.1%)
Unknown	155 (1.5%)
**Northern residence**	
Northern	1,308 (13.1%)
Southern	8,714 (86.9%)
**Known location of incident**	
Private residence	7,298 (72.8%)
Rooming house/other collective dwellings	596 (5.9%)
Other residential settings/shelters	229 (2.3%)
Other indoor space (non-hospital)	820 (8.2%)
Other (includes hospitals, transit, other)	229 (2.3%)
Outdoors	720 (7.2%)
Unknown	130 (1.3%)
**Number of substances directly involved in death**	
1	4,324 (43.1%)
2	4,790 (47.8%)
≥3	908 (9.1%)

*Multiple substances may have contributed to death, therefore these groups are not mutually exclusive.

### Combinations of substances involved in toxicity deaths

**Fig 1:**
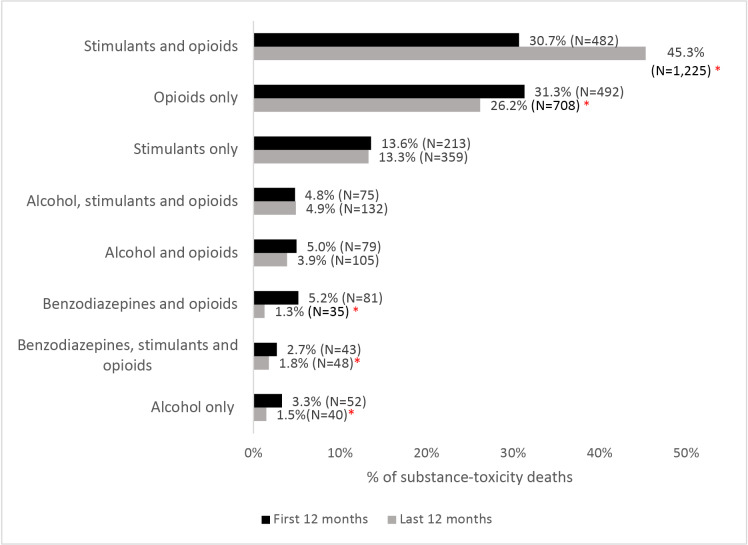
Proportion of substance-related toxicity deaths directly attributable to each substance combination, compared during the first 12 months and last 12 months of the study period. * indicates values of p < 0.05. Note: Categories <1% are not reported here due to space constraints.

We observed a 72.1% increase in the number of substance toxicity deaths from the first 12 months of the study period (N = 1,570) compared to the last 12 months (N = 2,702), and the combination of substances directly contributing to death changed considerably over this time (Fig 1). Notably, in the first 12 months of the study period, deaths involving opioids alone occurred most frequently (31.3% of all substance toxicity deaths; N = 492), followed by deaths involving both opioids and stimulants (30.7%, N = 482). However, the proportion of deaths involving both opioids and stimulants grew significantly, reaching 45.3% of deaths by the last 12 months of the study period (N = 1,225; p < 0.001). In parallel, the proportion of deaths involving opioids alone fell significantly over this period, reaching 26.2% by the last 12 months of the study (N = 708; p < 0.001). Although much less common, over the study period there were also significant declines in the prevalence of deaths attributed to alcohol only (3.3% to 1.5%; p < 0.001) and where benzodiazepine directly contributed to death in combination with either opioids (5.2% to 1.3%; p < 0.001), or both stimulants and opioids (2.7% to 1.8%; p = 0.04).

### Trends in rates of substance-related toxicity deaths

**Fig 2:**
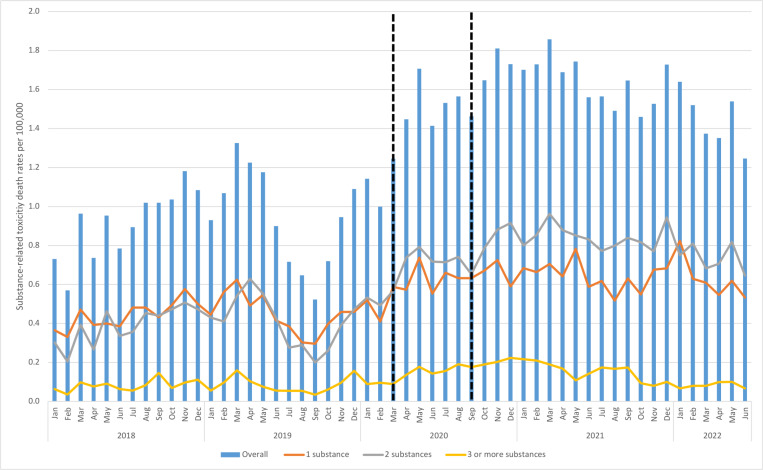
Monthly substance-related toxicity death rates (per 100,000 population), overall and stratified by the number of substances directly involved in death. ^*^Vertical bars represents the declaration of a pandemic-related state of emergency for COVID-19 and the additional intervention point identified using joinpoint models.

The monthly rate of substance-related toxicity deaths in Ontario rose throughout the study period, increasing 2-fold from 0.7 per 100,000 in January 2018 up to 1.4 per 100,000 in June 2022 (Fig 2). Using our selected ARIMA model (AIC = -14.60, BIC = -7.75), we observed a significant ramp increase in the rate of substance-related toxicities in March 2020 (+0.11 per 100,000 monthly, 95% CI: 0.01, 0.21 p = 0.03), followed by significant decrease in the trend beginning in September 2020 (-0.14 per 100,000 monthly, 95% CI: -0.23, -0.04, p = 0.01; Table 2).

**Table 2:** Rate of substance-related toxicities: results of the interventional ARIMA analyses.

ARIMA model (p,d,q)	Average monthly change in substance-related toxicities per 100,000 (95% CI)	p-value
**Overall analysis -** (0,1,2)_12_ no intercept		
March 2020 (ramp function)	+0.11 (0.01, 0.21)	0.03
September 2020 (ramp function)	-0.14 (-0.23, -0.04)	0.01

In total, 4,324 people (43.1%) who died from a substance-related toxicity death had one substance contributing to death, while 4,790 people had two substances contributing (47.8%) and 908 people (9.1%) had three or more substances directly involved in death. When stratified by the number of substances involved, three or more substances remained the least common throughout the study period, but monthly rates still rose over the four year period (from 0.06 up to 0.07 per 100,000 from January 2018 to June 2022) (Fig 2). In contrast, deaths involving one or two substances were more common. While the rate of single-substance deaths increased 25% (from 0.4 to 0.5 per 100,000 from January 2018 to June 2022), the rate of deaths involving two substances increased nearly 2.5 fold (from 0.3 to 0.7 per 100,000 from January 2018 to June 2022) and was the most common category by the end of study period.

### Prior healthcare encounters for substance-related toxicity and substance use disorder

Approximately one-fifth of individuals (19.9%; N = 1,994) were treated in a hospital setting for a substance-related toxicity involving opioids, stimulants, benzodiazepines and/or alcohol in the year prior to death (Table 3). The majority of these toxicities were opioid-related (17.4%; N = 1,748), followed by stimulant-related toxicities (5.0%; N = 499), benzodiazepine-related toxicities (2.0%; N = 204) and alcohol-related toxicities (1.0%; N = 103). Additionally, 60.9% of people who died from a substance-related toxicity (N = 6,098) had a substance use disorder diagnosis (any type) at time of death. Specifically, almost one-quarter of people had a hospital visit relating to opioid use disorder (23.2%; N = 2,323), stimulant use disorder (22.1%; N = 2,219) and/or alcohol use disorder (24.2%; N = 2,429) in the past five years; whereas hospital visits related to benzodiazepine use disorder diagnoses were much less common (2.5%; N = 252). Similarly, over one-third of people had an outpatient physician visit related to a substance use disorder in the past year (excluding alcohol use disorder) (36.6%; N = 3,671), with 5.4% (N = 537) having an outpatient visit relating to alcohol use disorder.

**Table 3:** Previous healthcare encounters among individuals who died from a substance-related toxicity.

	N (%)
**Hospital treated substance-related toxicities** (prior 1 year)	1,994 (19.9%)
Opioid-related toxicities^*^	1,748 (17.4%)
Stimulant-related toxicities^*^	499 (5.0%)
Benzodiazepine-related toxicities^*^	204 (2.0%)
Alcohol-related toxicities^*^	103 (1.0%)
**Any healthcare encounter for substance use disorder** (prior 5 years for hospital based and 1 year for outpatient diagnoses)*	6,098 (60.9%)
**Hospital based encounters with substance use disorder diagnoses** (any diagnosis type; prior 5 years)	4,670 (46.6%)
Opioid use disorder	2,323 (23.2%)
Stimulant use disorder	2,219 (22.1%)
Benzodiazepine use disorder	252 (2.5%)
Alcohol use disorder	2,429 (24.2%)
**Hospital based encounters with substance use disorder diagnoses** (most responsible diagnosis; prior 5 years)	3,516 (35.1%)
Opioid use disorder	1,518 (15.1%)
Stimulant use disorder	1,248 (12.5%)
Benzodiazepine use disorder	141 (1.4%)
Alcohol use disorder	1,756 (17.5%)
**Outpatient Diagnoses** (prior 1 year)	
Alcohol use disorder outpatient diagnoses	537 (5.4%)
Any other substance use disorder outpatient diagnoses	3,671 (36.6%)

*Multiple substances may have contributed to death, therefore these groups are not mutually exclusive.

## Discussion

Between January 2018 and June 2022, more than 10,000 accidental substance toxicity deaths were reported across Ontario, with the absolute number of deaths almost doubling between the first and last year of our study period. This is equivalent to approximately six deaths each day during this period due to substance-related toxicities across Ontario. Further, we found that the COVID-19 pandemic led to a significant temporary acceleration in rates of substance-related toxicity deaths. Specifically, there was a trend towards multiple substance involvement in deaths that was primarily driven by higher frequency of toxicities involving use of both opioids and stimulants, with deaths involving alcohol and benzodiazepines as direct contributors remaining less common. Overall, many individuals had visits related to substance use disorder in the five years prior to death and were treated in a hospital setting for a substance-related toxicity year prior to death. These prior encounters with the healthcare system represent missed windows of opportunity for healthcare professionals to engage and support people who use drugs [35].

The initial increase in substance-related toxicity deaths we observed following the COVID-19 pandemic-related state of emergency may be attributable to widespread disruptions in access to healthcare services that occurred across Ontario early in the pandemic [36,37]. Access to mental health-related services and harm reduction programs were particularly limited [38–40], as many in-person supports were paused, operated at reduced capacity or were temporarily closed. Furthermore, pandemic-related public health measures, such as lockdowns, physical distancing, and reduced social interactions, likely contributed to heightened isolation and stress, leading to changing patterns of substance use [4,5]. At the same time, changes in supply chains due to border closures may have resulted in a more volatile and unpredictable unregulated drug supply [41], increasing the risk of fatal toxicities. Interestingly, we observed a reversal in this trend with a gradual decrease in substance toxicity deaths beginning in September 2020. This broadly aligns with the staged reversal of pandemic-related public health measures across some regions in Ontario [42], and suggests that although significant increases in mortality occurred initially this was not sustained throughout the entirety of the pandemic. Future research is needed to understand how the combination of reduced healthcare access, and a more toxic drug supply contributed to the initial rise in toxicity deaths during the pandemic.

Our findings also contribute to the emerging body of literature highlighting that the use of multiple substances and the increasing adulteration of the unregulated opioid supply with non-opioid substances are resulting in increasing rates of harms related to substance use [18,43]. In particular, we found that opioids and stimulants combined made up the highest proportion of substance-related toxicity deaths, with the absolute number of deaths more than doubling over the study period. This aligns with trends reported across the US where deaths involving both cocaine and opioids increased 46.0% per year between 2014–2017 [44]. Similarly, a recent study in Quebec, Canada found that the proportion of opioid-related deaths involving cocaine increased from 24% to 51% between 2016 and 2021, with methamphetamine detected in 41% of deaths by 2021 [45]. Qualitative evidence also indicates that people often use opioids in combination with stimulants to combat the sedating effects of the unregulated opioid supply as a means of staying alert [16]. However, suppressing the depressant effects of opioids with stimulants may lead to a false impression of tolerance and more frequent use of opioids, which can increase overdose risks [46]. This is especially concerning as traditional approaches for reversing opioid toxicities such as naloxone, do not reverse the effects of stimulants or other non-opioid sedatives (e.g., benzodiazepines). Furthermore, there are currently no pharmaceutical options accessible at the community level to reverse the effects of non-opioid substances [47]. This further reinforces the need for harm reduction strategies and treatment options to reflect the growing combinations of substances that people are exposed to. Harm reduction strategies may include the provision of oxygen during an overdose response, providing naloxone to individuals who primarily use stimulants as research shows these individuals may be more likely to witness overdoses[48], and OAT programs which better address multiple risks including exposure to stimulants and management of benzodiazepine withdrawal.

Importantly, we also found that approximately one in five people had been treated for a non-fatal substance-related toxicity in the year before their death, with the majority of these incidents being opioid-related. This represents an increase from a previous study conducted in Ontario, which found that less than 10% of individuals who died from an opioid toxicity between 2015 and 2016 had a prior hospital-treated toxicity [49]. Therefore, these hospital visits increasingly represent important opportunities to provide care to people who use substances, including treatment initiation for those with a substance use disorder diagnosis and coordination of connections with harm reduction, substance use treatment and primary care services upon discharge [50]. However, transitions in care between acute hospital care settings to community-based care can vary greatly [35] given that people who use substances face additional barriers and stigma when accessing care in both settings [51]. Prior research has found that only one in eighteen people (4.1%) were dispensed OAT within a week of discharge for a hospital treated opioid-related toxicity in Ontario [52], despite current clinical guidelines recommending that OAT be offered to all patients with OUD when in hospital [53]. However, we also found that two in five substance-related toxicity deaths occurred among people without a recorded substance use disorder diagnosis, reinforcing that not all people at risk of harm from substance use have such a diagnosis, and therefore may not be eligible for treatment. As a result, heightened access to harm reduction programs and services that extend beyond treatment - such as supervised consumption sites and prescribed safer opioid supply programs - is needed to ensure widespread prevention efforts for accidental toxicities among all people at risk of these harms.

### Limitations

A strength of this study includes the use of a novel database to provide insights into the trends of substance-related toxicity deaths across Ontario. However, a few limitations warrant discussion. First, we excluded individuals who did not have a valid Ontario health card (approximately 7% of all deaths) to allow for linkage to healthcare records. Therefore, these analyses are an underestimate of the full burden of substance toxicity deaths in Ontario over the study period and the generalizability of our findings may be limited to people who are disconnected from the healthcare system. Second, we only included confirmed deaths where alcohol, stimulants, benzodiazepines, or opioids directly contributed to death. It is possible that suspected substance toxicity deaths may be later determined by the investigating coroner to be related to the above substances, and these were not included in this study. Data from the Chief Coroner of Ontario on drug toxicity deaths are received only for death investigations that are nearly complete and as such we anticipate the potential underestimation of death rates in our study is minimal. Third, there is no validated definition of substance use disorders using health administrative data. Therefore, we relied on hospital records over a five year period, and physician visits in the one year prior to death to identify people with a likely active substance use disorder at time of death. However, it is possible that misclassification exists in this definition. Fourth, we only include benzodiazepine toxicity deaths where a benzodiazepine was identified as a direct contributor to death. Because thresholds are not well established for benzodiazepine toxicity in post-mortem toxicology, our findings may not accurately reflect the presence of benzodiazepine detection in substance toxicity deaths, which has reached over 45% in other studies of opioid toxicity deaths in Ontario during the COVID-19 pandemic [54]. Fifth, the use of seasonal differencing in our ARIMA model to achieve stationarity may complicate the interpretation of trend components, as it removes the seasonal pattern from the data. This can make it more difficult to distinguish between long-term trends and short-term fluctuations. Finally, our reliance on healthcare administrative data limited our ability to capture some pandemic-related factors such as stress, job loss, and housing instability, while the joinpoint model, though effective for detecting trend shifts, may miss gradual or complex changes and does not account for confounding variables. These limitations may affect the generalizability of our findings, as the observed trends may not reflect the experiences of people disproportionately impacted by the pandemic. Future research could benefit from integrating complementary data sources such as surveys or interviews to help capture broader social impacts.

## Conclusions

The number of accidental substance toxicity deaths almost doubled between January 2018 and June 2022, highlighting the growing loss of life from substance-related toxicities across Ontario and the increasing involvement of multiple substances including both opioids and stimulants in these deaths. This continued to worsen following the declaration of the COVID-19 pandemic state of emergency, with a high frequency of hospital interactions observed prior to death. This suggests the need for improved coordination of care throughout the healthcare system to meet the complex needs of people who use drugs and the importance of enhanced access to mental health and harm reduction services in future public health crises.

## Supporting information

S1 Table Definitions of prior substance-related toxicities in the year prior to death, using emergency department (ED) visits or in-patient hospitalizations. (DOCX)

S2 Table Definitions of substance use disorders. (DOCX)

S3 Table Potential ARIMA model AIC and BIC values. (DOCX)
