# Supplementary material for: The changing role of substances: trends, characteristics of individuals and prior healthcare utilization among individuals with accidental substance-related toxicity deaths in Ontario Canada
Source: PLoS One. 2025 May 23;20(5):e0324732. doi: 10.1371/journal.pone.0324732 (PMC12101627; doi:10.1371/journal.pone.0324732)
Supplement: S1 Table — (DOCX) [file pone.0324732.s001.docx]

**S1 Table:** Definitions of prior substance-related toxicities in the year prior to death, using emergency department (ED) visits or in-patient hospitalizations

| We identified any emergency department visits (NACRS) or in-patient hospitalizations (DAD) for a non-fatal substance-related toxicity in the 1-year before death (excluding date of death), defined using the following ICD-10 codes separately for:  **Opioid-related toxicity**   - ICD-10 codes: T400, T401, T402, T403, T404, T406 |
| --- |
| **Stimulant-related toxicity**   - ICD-10 codes: T436, T405 |
| **Benzodiazepine-related toxicity**   - ICD-10 code: T424 |
| **Alcohol-related toxicity**   - ICD-10 code: T510 |
| **Substance-related toxicity**  Using the definitions for the above substance-specific toxicity events, determine if a person had ≥1 hospital treated substance-related toxicity in the year prior to death |
