# Supplementary material for: The changing role of substances: trends, characteristics of individuals and prior healthcare utilization among individuals with accidental substance-related toxicity deaths in Ontario Canada
Source: PLoS One. 2025 May 23;20(5):e0324732. doi: 10.1371/journal.pone.0324732 (PMC12101627; doi:10.1371/journal.pone.0324732)
Supplement: S2 Table — (DOCX) [file pone.0324732.s002.docx]

**S2 Table**: Definitions of substance use disorders.

| **Substance** | **Classification** | **Defined as:** |
| --- | --- | --- |
| **Alcohol** | Alcohol use disorder hospital diagnoses only (using data from DAD, NACRS & OMHRS databases) in the last 5 years | **ICD-10 (DAD/NACRS/OMHRS):**   - F10 - K70 - G312 - G621 - G721 - I426 - K292 - K860 - Z502 - Z714 - Z8640 - E244 - K852   **ICD-9 (OMHRS):**   - 291.x [excl. 291.82] - 303.x - 305.0 |
|  | **Alcohol use disorder** outpatient definition | In prior 1 year for alcohol use disorder outpatient visits (OHIP DXCODE: 303, 291) |
| **Opioids** | Opioid Use Disorder hospital diagnoses only (using data from DAD, NACRS & OMHRS databases) | In the past 5 years, any hospital opioid use disorder diagnosis using:  **ICD-10 (DAD/NACRS/OMHRS):**   - F11: Opioid-related disorders   **ICD-9 (OMHRS):**   - 304.0 - 305.5 |
|  | Opioid use disorder outpatient | Any previous opioid-agonist related outpatient visits (OHIP) in year prior to death were identified using fee codes: K682, K683 & K684 |
| **Stimulants** | **Stimulant use disorder (hospital diagnoses; using data from DAD, NACRS & OMHRS databases).** | **In the past 5 years any hospital diagnosis using:**  **ICD-10 (DAD/NACRS/OMHRS):**   - **F14** - **F15**   **ICD-9 (OMHRS):** **304.4** |
| **Benzodiazepines** | Benzodiazepine use disorder (hospital diagnoses; using data from DAD, NACRS & OMHRS databases) | **In the past 5 years any hospital diagnosis of:**  **ICD-10 (DAD/NACRS/OMHRS):**   - **F13**   **ICD-9 (OMHRS):**   - **304.1** |
| **Any substance use** | Any substance use disorder diagnosis | Defined as hospital-based encounters with substance use disorder diagnoses in the prior 5 years or substance use disorder outpatient diagnoses in the prior 1 year (not including alcohol use disorder outpatient diagnoses) |
|  | Any other substance use disorder related outpatient visits | Any outpatient visit for a substance us disorder (with the exception of alcohol use disorder outpatient diagnoses, they are not included in this definition) in the previous year |
