# Supplementary material for: The changing role of substances: trends, characteristics of individuals and prior healthcare utilization among individuals with accidental substance-related toxicity deaths in Ontario Canada
Source: PLoS One. 2025 May 23;20(5):e0324732. doi: 10.1371/journal.pone.0324732 (PMC12101627; doi:10.1371/journal.pone.0324732)
Supplement: S3 Table — (DOCX) [file pone.0324732.s003.docx]

**S3 Table:** Potential ARIMA Model AIC and BIC Values

| **ARIMA model (p,d,q)** | **AIC Value** | **BIC Value** |
| --- | --- | --- |
| (0,1,2)_12_ no intercept | -14.60 | -7.75 |
| (0,1,2) no intercept | -34.97 | -27.09 |
| (9,1,1)_12_ no intercept | -3.86 | 16.70 |
| (9,1,1) no intercept | -26.75 | -3.10 |
